# The role of mitochondria in the gut-kidney axis: implications for kidney health

**DOI:** 10.3389/fphar.2026.1851705

**Published:** 2026-06-18

**Authors:** Qin Hu, Hua Jin, Lan Hu, Xu Li

**Affiliations:** 1 The First Affiliated Hospital, Anhui University of Chinese Medicine, Hefei, China; 2 Department of Nephrology, The First Affiliated Hospital, Anhui University of Chinese Medicine, Hefei, Anhui, China; 3 Center for Xin’an Medicine and Modernization of Traditional Chinese Medicine of IHM, The First Affiliated Hospital of Anhui University of Chinese Medicine, Hefei, Anhui, China

**Keywords:** chronic kidney disease, gut microbiota, gut-kidney axis, kidney disease, mitochondria

## Abstract

Mitochondria, multifunctional organelles that regulate cellular energy metabolism and signaling pathways, play a pivotal role in maintaining the physiological functions of the gut and kidneys, as well as influencing the progression of chronic kidney disease (CKD). Through the gut-kidney crosstalk, gut microbiota modulate gut and renal pathophysiology and also influence mitochondrial activity in intestinal and renal cells. This review explores the regulatory roles of mitochondria in preserving epithelial barrier integrity, regulating intestinal metabolism, and maintaining gut microbiota homeostasis. It also examines the contributions of mitochondrial biogenesis, dynamics, autophagy abnormalities, and mitochondrial DNA (mtDNA) damage to renal pathological progression. Moreover, we highlight the bidirectional interactions between intestinal and renal mitochondria via the microbiota-mitochondria-kidney axis and mechanisms involving inflammation, oxidative stress, and ferroptosis. Therefore, targeting mitochondrial regulation through non-pharmacological interventions such as dietary adjustments, probiotic supplementation and fecal microbiota transplantation (FMT) emerges as a promising therapeutic strategy for maintaining renal health by optimizing mitochondrial function. In conclusion, elucidating the mechanisms of mitochondrial involvement in the gut-kidney axis will lay the foundation for novel therapeutic approaches to CKD and other gut-kidney axis-related disorders.

## 1 Introduction

Mitochondria are highly dynamic, multifunctional organelles in eukaryotic cells responsible for converting nutrients that include glucose, fatty acids, and amino acids into adenosine triphosphate (ATP) through aerobic respiration, earning them the designation as powerhouses of the cell (Song et al., 2021). Mitochondria exert a pivotal regulatory role in regulating metabolism, generating reactive oxygen species (ROS), controlling apoptosis and autophagy, maintaining intracellular calcium homeostasis, and thermogenesis (Che et al., 2014; Brooks and Dong, 2007). The kidney counts among the organs with the highest energy requirements, trailing only the heart in terms of mitochondrial density and oxygen utilization (Pagliarini et al., 2008). Kidney-related physiological processes including filtration, tubular reabsorption, and acid–base regulation are highly dependent on ATP generated by mitochondria. Evidence indicates that acute or chronic kidney injury can trigger mitochondrial dysfunction, initiating a cascade characterized by impaired Na^+^/K^+^-ATPase activity, energy depletion, excessive ROS accumulation, activation of mitochondrial apoptotic pathways, and amplification of renal and systemic inflammatory responses. Collectively, these events accelerate progression toward irreversible fibrosis (P and Ph, 2017). Mitochondrial impairment also contributes to myofibroblast activation and extracellular matrix deposition, driving tubulointerstitial fibrosis, a pathological hallmark of CKD progression (Srivastava et al., 2020). Accordingly, abnormalities in mitochondrial biogenesis, dynamics, and mitophagy, nuclear DNA (nDNA) and mitochondrial DNA (mtDNA) that impair mitochondrial function, bear a strong correlation with the pathogenesis of kidney diseases (Zhang et al., 2023).

The intestinal tract serves as the largest metabolic and immunological organ in the human body. Its digestive and absorptive functions, together with microbial metabolic activities, provide energy substrates and functional regulatory molecules for mitochondria. The renewal of intestinal epithelial cells (IECs), the synthesis of tight junction proteins, and the secretion of antimicrobial peptides all depend on mitochondrial ATP supply; meanwhile, the physiologically hypoxic intestinal lumen maintained by oxygen consumption during mitochondrial oxidative phosphorylation (OXPHOS) serves as a key ecological niche for suppressing the overproliferation of facultative anaerobes (Rodríguez-Colman et al., 2017). Therefore, mitochondria are not only the central hub of cellular energy metabolism but also the molecular foundation for maintaining physiological homeostasis in the intestine and kidneys.

In recent years, the establishment of the “gut-kidney axis” concept has revealed a bidirectional regulatory relationship between the gut microbiota and kidney disease. Multiple gut microbiota metabolites, including short-chain fatty acids (SCFAs) and trimethylamine N-oxide (TMAO), have been demonstrated to regulate key transcription factors and enzymes associated with mitochondrial biogenesis, metabolism, and oxidative stress, thereby influencing renal function (He et al., 2024; Lee et al., 2024). Beneficial metabolites including butyrate and indole-3-propionic acid (IPA) effectively protect renal mitochondrial function and mitigate kidney injury (Shi et al., 2025; Zeng et al., 2025). In contrast, dysbiosis disrupts the intestinal barrier and increases permeability (leaky gut), facilitating translocation of deleterious products such as lipopolysaccharide (LPS) and indoxyl sulfate (IS) into the circulation and ultimately to the kidney. These metabolites impair renal metabolic capacity by inhibiting the mitochondrial respiratory chain, disrupting mitophagy, and aggravating mitochondrial oxidative stress (Mutsaers et al., 2013). Research indicates that declining renal function and accumulation of uremic toxins induce electrolyte disturbances, metabolic acidosis, and heightened inflammatory signaling, which further exacerbate gut dysbiosis (Cao et al., 2022; Nishiyama et al., 2019). Thus, mitochondria are deeply involved in the bidirectional communication of the gut–kidney axis by regulating the physiological homeostasis of both the intestine and kidneys.

Recent evidence suggests that mitochondrial dysfunction represents a critical molecular event bridging dysbiosis of the gut microbiota and kidney disease (Cao et al., 2022),positioning mitochondrial regulation within the gut–kidney axis as a frontier area in interdisciplinary research. Although prior studies have examined interactions between gut-derived metabolites and mitochondrial function, as well as the role of mitochondrial dysfunction in renal pathology, comprehensive syntheses detailing how mitochondria mechanistically connect the gut and kidney remain limited. Studies on the gut-kidney axis have long focused on the forward pathway of gut microbiota dysregulation - uremic toxin accumulation - renal injury, whereas the molecular mechanisms underlying the reverse feedback of accumulated uremic toxins in chronic kidney disease (CKD) affecting the intestine and other distal organs remain insufficiently understood. CKD is highly heterogeneous in etiology and disease progression, and current evidence is largely restricted to diabetic kidney disease (DKD) and ischemia/reperfusion-induced acute kidney injury (I/R-AKI) models. To date, there is a lack of systematic comparison and mechanistic characterization regarding the etiology-specific differences in mitochondria-mediated gut-kidney axis regulation among other CKD subtypes, including hypertensive nephrosclerosis, autosomal dominant polycystic kidney disease (ADPKD), and lupus nephritis. In this review, we systematically summarize the roles of intestinal mitochondria in barrier integrity, metabolism, and immune regulation. Then, we discuss from the perspective of mitochondria how the dysregulation of mitochondrial biogenesis, dynamics, mitophagy, and DNA damage affects the development and progression of acute and chronic kidney diseases. We further highlight major signaling pathways underpinning gut–kidney crosstalk, with emphasis on the microbiome metabolite–mitochondria–kidney axis, inflammation–oxidative stress pathways, and ferroptosis-related mechanisms. This review systematically integrates the cascade crosstalk among microbial metabolites, mitochondrial signaling, mitochondrial quality control, and the kidney, and proposes a closed-loop regulatory mechanism of the mitochondria–gut–kidney axis. Finally, we review emerging therapeutic strategies targeting mitochondrial regulation within the gut–kidney axis, encompassing probiotic supplementation, dietary interventions, and FMT. Collectively, mitochondria act as a pivotal mediator linking intestinal dysbiosis to renal pathology; advancing mechanistic understanding of these processes may provide novel targets for the prevention and treatment of kidney diseases.

## 2 Mitochondria serve as the regulatory hub for maintaining gut health

### 2.1 Maintenance of intestinal barrier integrity

The intestinal barrier is composed of a single layer of epithelial cells, tight junctions, a mucus layer, and antimicrobial peptides. Barrier integrity relies on the continuous renewal of intestinal epithelial cells (IECs) and an adequate energy supply. Mitochondria support IEC proliferation, migration, and differentiation primarily by generating ATP via oxidative phosphorylation (OXPHOS), thereby sustaining epithelial turnover and barrier homeostasis (Guerbette et al., 2022). Intestinal stem cells (ISCs) depend on mitochondrial fatty acid β-oxidation (FAO) to maintain stemness, whereas Paneth cells (PCs) preferentially utilize glycolysis and produce lactate, which can be used as a metabolic substrate to fuel OXPHOS in adjacent Lgr5^+^ ISCs (Gassler, 2017). deficiency of the mitochondrial chaperone Hsp60 activates the unfolded protein response (UPR), compromises mitochondrial function, and reduces ISC proliferative capacity; concomitantly, Paneth cell granule secretion is impaired, resulting in defective epithelial renewal and diminished barrier repair (Khaloian et al., 2020). Additionally, Mitochondria also regulate epithelial apoptosis, which is required to remove damaged or aberrant cells and maintain physiological turnover. At physiological levels, mitochondrial ROS (mtROS) can activate p38 MAPK signaling to promote IEC differentiation and crypt formation while supporting epithelial survival (Rodríguez-Colman et al., 2017). However, under high-fat diet (HFD) or inflammatory conditions, excessive mtROS damages mtDNA and ETC., complexes, triggering the Bcl-2-associated X protein (Bax)/ Bcl-2 antagonist killing protein (Bak)-mediated mitochondrial outer membrane permeabilization (MOMP) apoptotic pathway, leading to excessive epithelial cell apoptosis (Li and Li, 2020). ROS overload can trigger a decrease in mitochondrial membrane potential (ΔΨm), releasing large amounts of cytochrome C, which leads to mitochondrial damage and accelerates epithelial cell apoptosis (Salimi et al., 2020). Studies reveal that elevated mtROS in ulcerative colitis (UC) patients is associated with reduced mitochondrial acetyl-CoA thiolase activity, and mucosal biopsies demonstrate significantly increased IEC apoptosis (Santhanam et al., 2007) The epithelial layer exhibits discontinuities due to massive cell apoptosis, creating pathways for microbial flora and toxins to enter the bloodstream, resulting in intestinal permeability. Tight junction proteins between IECs serve as the barrier preventing small molecular toxins such as IS and LPS from entering the blood circulation. A high-fat diet (HFD) induces mitochondrial swelling in colonic epithelial cells, reduces, ETC., complex II/III activity, and decreases ATP production, leading to downregulation of tight junction proteins occludin and ZO-1 and increased intestinal permeability (Guerbette et al., 2025). Conversely, activation of mitophagy can attenuate IEC apoptosis, reduce hyperpermeability, and ameliorate intestinal injury (Liu et al., 2017). In addition, goblet cells secrete mucins that form the mucus barrier. Mitochondrial dysfunction can limit ATP availability and impair MUC2 synthesis, enabling bacteria to adhere directly to the epithelial surface and release cytotoxic factors. In a citrinin (CTN)-induced intestinal injury model, mitochondrial pathway activation was associated with reduced MUC2 expression and increased epithelial apoptosis (Li et al., 2024). Moreover, MUC2-deficient mice exhibit increased intestinal permeability, defects in intercellular junction structures, mitochondrial damage, and reduced intestinal ATP content, ultimately leading to leaky gut syndrome (Borisova et al., 2020). Intestinal epithelial cell renewal and metabolism, desmin synthesis, and mucin secretion are all closely linked to mitochondrial function. Impaired mitochondrial function increases intestinal permeability, allowing endotoxins and microbial metabolites to enter the bloodstream, thereby triggering renal injury.

### 2.2 Regulation of gut microbiota homeostasis

The gut microbiota and host mitochondria constitute a bidirectional regulatory network. Mitochondria modulate microbial composition by regulating oxygen concentration and metabolic byproducts within the gut, while microbial metabolites in turn regulate mitochondrial function, jointly maintaining intestinal homeostasis.

In the colonic microenvironment, epithelial cells consume oxygen via mitochondrial OXPHOS, thereby maintaining a physiologically hypoxic lumen that restrains the expansion of facultative anaerobes. Microbial metabolites including butyrate can activate PPAR-γ signaling within colonocytes, further enhancing mitochondrial OXPHOS and reinforcing epithelial oxygen consumption. This oxygen-scavenging program suppresses the overgrowth of potentially pathogenic Enterobacteriaceae, including *Escherichia coli* and *Salmonella*, and helps preserve microbial homeostasis (Byn et al., 2017). Conversely, mitochondrial dysfunction reduces epithelial oxygen utilization, elevates luminal oxygen availability, and promotes the bloom of facultative anaerobic bacteria, precipitating dysbiosis. Studies indicate that in high-fat diet (HFD)-fed mice, decreased mitochondrial, ETC., complex activity in colonic tissue correlates significantly with increased Enterobacteriaceae abundance, elevated luminal oxygen and nitrate levels, and enhanced *E. coli* proliferation (Yoo et al., 2021). In addition, Paneth cell packaging and secretion of antimicrobial peptides depend on mitochondrial ATP production and Rab8a-mediated vesicular trafficking; mitochondrial injury compromises antimicrobial peptide release and weakens the host capacity to limit pathogen colonization (Adolph et al., 2013). Thus, mitochondria shape and stabilize the gut microbiota ecosystem through a tri-axial linkage of energy-hypoxia-antimicrobial peptides.

Microbiota-derived metabolites further influence microbial ecology by reprogramming epithelial mitochondrial metabolism. Butyrate, a preferred fuel for colonocytes, is converted via mitochondrial fatty acid oxidation (FAO) into acetyl-CoA, supporting epithelial energetics and exerting epigenetic effects, in part through inhibition of histone deacetylase (HDAC) activity (Donohoe et al., 2011). Microbial lactate can signal through GPR81 to stimulate Paneth cell Wnt production, thereby supporting ISCs proliferation and indirectly shaping microbial community structure (Lee et al., 2018). Moreover, indole derivatives produced during tryptophan metabolism activate aryl hydrocarbon receptor (AHR) signaling, enhance mitochondrial function, promote IL-22 production, and contribute to microbial homeostasis (Scott et al., 2020). Conversely, harmful metabolites like hydrogen sulfide (H_2_S) inhibit mitochondrial function, creating a vicious cycle. H_2_S suppresses, ETC., complex IV activity, reduces ATP production, lowers epithelial oxygen consumption, and promotes sulfate-reducing bacteria growth (Wlodarska et al., 2017).

### 2.3 Mediation of intestinal immunity and inflammatory balance

Mitochondria exert a dual role in regulating intestinal immune responses. On one hand, their metabolic byproducts (such as ROS, ATP, and mtDNA) serve as signaling molecules that activate immune cells or induce inflammatory reactions. On the other hand, mitochondria also participate in establishing and maintaining immune tolerance by regulating IEC metabolism and stem cell function. At physiological levels, mtROS can enhance mucosal defense by activating NF-κB signaling, promoting antimicrobial peptide expression, and facilitating immune cell recruitment (Formentini et al., 2017). However, severe mitochondrial dysfunction leads to excessive ROS production and mtDNA release, which act as damage-associated molecular patterns (DAMPs) to activate NLRP3 inflammasomes and the cGAS–STING pathway, driving secretion of proinflammatory cytokines such as IL-1β and IL-18 and amplifying inflammation (Boy et al., 2018; González-Bosch et al., 2021). Mitochondria maintain tolerance toward symbiotic bacteria by regulating immune signaling in epithelial cells. In IEC cells, TLR4 recognizes LPS from the microbiota and regulates, ETC., activity via the mitochondrial TRAF6-ECSIT signaling pathway, limiting mtROS production and preventing excessive inflammation (West et al., 2011). Additionally, mitochondrial metabolites (succinate and NAD^+^) serve as immunometabolic cues influencing cytokine production, including IL-22 and IL-10. IL-22 promotes epithelial proliferation and repair by enhancing mitochondrial function through STAT3 activation, whereas IL-10 suppresses inflammation by inhibiting glycolysis, supporting OXPHOS, and promoting mitophagy (Ip et al., 2017; Zha et al., 2019). Aromatic hydrocarbon receptor (Ahr), derived from microbial tryptophan metabolism, can enter mitochondria to stabilize the respiratory chain complexes. This synergistically maintains the functions of Treg and ILC3, promoting the resolution of inflammation (Metidji et al., 2018).

**FIGURE 1:**
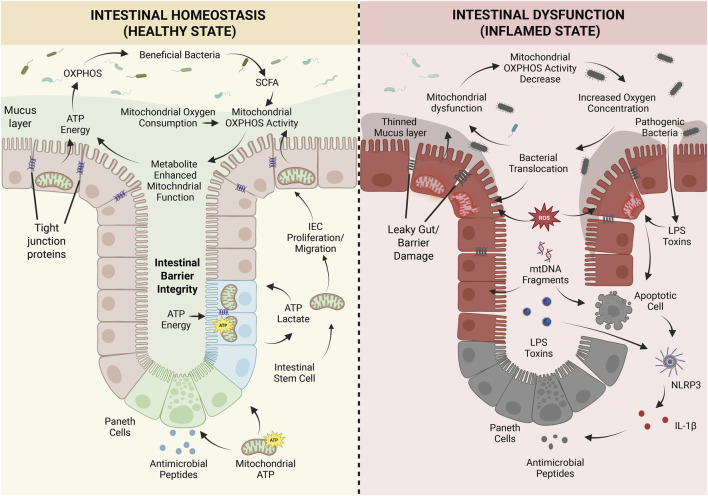
Mitochondrial orchestration of intestinal barrier homeostasis and the pathological mechanisms of mitochondrial dysfunction. (Left) Physiological State: Functional mitochondria support epithelial integrity (tight junctions, mucus) and maintain a hypoxic niche for commensal microbiota via efficient ATP production and oxygen consumption. These bacteria produce short-chain fatty acids (SCFAs), which feedback to enhance mitochondrial function. (Right) Pathological State: Mitochondrial dysfunction drives ATP depletion, ROS accumulation, and mtDNA release. These events trigger tight junction disruption (Leaky Gut), dysbiosis, and activation of inflammatory cascades. This cascade triggers IEC apoptosis, bacterial translocation, and the infiltration of LPS and toxins, thereby amplifying the inflammatory response.

Table 1 illustrates the impact of mitochondrial dysfunction on intestinal function across different models. Collectively, mitochondria act as key mediators of intestinal homeostasis through three interrelated processes: bioenergetic support, ecological conditioning of the luminal niche, and immunometabolic regulation. Under physiological conditions, mitochondrial OXPHOS supplies ATP to sustain epithelial renewal and barrier integrity, while epithelial oxygen consumption and antimicrobial peptide secretion help maintain microbial community stability. Simultaneously, mitochondria function as an immunometabolic node that balances mucosal tolerance and host defense. Under pathological conditions, mitochondrial dysfunction triggers ATP depletion, ROS surges, and inflammatory signaling, promoting barrier disruption, dysbiosis, and local inflammation (Figure 1). These intestinal disturbances extend beyond the gut by increasing systemic exposure to endotoxins and harmful microbial metabolites, thereby contributing to renal injury.

**TABLE 1:** Summary of the impact of mitochondrial dysfunction on intestinal function.

Study type	Disease/ Model type	Type of mitochondrial injury/ Pathological change	Impact on intestinal function	References
Clinical Studies	Ulcerative Colitis (UC)	Reduction in mitochondrial acetoacetyl-CoA thiolase;	Increased apoptosis of IECs	Santhanam et al. (2007)
Animal Studies	High-Fat Diet (HFD)	Reduced ATP production; reduced activity of, ETC., complexes II/III	Downregulation of tight junction proteins (Occludin, ZO-1)	Guerbette et al. (2025)
​	High-Fat Diet (HFD)	Damage to mtDNA and ETC., complexes	Activation of the Bax/Bak mitochondrial apoptosis pathway, epithelial cell apoptosis	Li and Li (2020)
​	High-Fat Diet (HFD)	Decreased mitochondrial, ETC., complex activity; reduced epithelial oxygen consumption	Increased luminal oxygen and nitrate levels promote the proliferation of facultative anaerobes	Yoo et al. (2021)
​	Citrinin (CTN)-induced Intestinal Injury	Activation of the mitochondrial apoptotic pathway	Decreased MUC2 protein expression triggers cell apoptosis	Li et al. (2024)
​	MUC2 Knockout	Mitochondrial injury; morphological defects in intercellular junctions	Reduced intestinal ATP levels lead to leaky gut syndrome	Borisova et al. (2020)
*In vitro*	Mitochondrial Chaperone (Hsp60) Deficiency	Induction of UPR activation; mitochondrial dysfunction	Decreased proliferation of ISCs; impaired secretion in Paneth cells	Khaloian et al. (2020)
​	High-concentration H_2_S Exposure	Reduced ATP production; Inhibition of, ETC., complex IV activity	decreased epithelial oxygen consumption	Wlodarska et al. (2017)
​	Severe Inflammation/ Mitochondrial Injury	Excessive ROS production; mtDNA damage and release	Release of DAMPs activates NLRP3 and cGAS-STING pathways, amplifying inflammation	Boy et al. (2018), González-Bosch et al. (2021)

## 3 Mitochondria are key organelles regulating kidney function

### 3.1 Maintenance of normal renal physiology

Renal cells are rich in mitochondria, which account for approximately 30% of the cell volume. Tubular epithelial cells demand substantial quantities of ATP for sustaining solute reabsorption. Mitochondria meet this demand by generating ATP via OXPHOS, thereby sustaining the activity of ATP-dependent transporters such as Na^+^/K^+^-ATPase and providing the energetic basis for active tubular transport (Soltoff, 1986). Although glomerular filtration is largely a passive process driven by intraglomerular hydrostatic pressure, mitochondria remain essential for maintaining stable renal hemodynamics and preserving podocyte structure, which together ensure the integrity of the filtration barrier (Holechek, 2003). Furthermore, In nephron segments such as the loop of Henle and distal tubules, mitochondrial energy supply is also critical for urine concentration/dilution and electrolyte homeostasis. In addition, mitochondria contribute to renal glucose handling by regulating pyruvate entry into the tricarboxylic acid (TCA) cycle and by metabolizing lactate (Chen et al., 2017). They further support renal lipid metabolism: in proximal tubules, mitochondrial β-oxidation of fatty acids not only provides energy but also limits intracellular lipid accumulation, thereby reducing lipotoxic injury (Console et al., 2020). Accordingly, mitochondrial dysfunction or structural damage can compromise filtration, reabsorption, and repair, manifesting clinically as proteinuria, nocturia, and progressive loss of renal function (Pavlović et al., 2025).

### 3.2 Mitochondrial damage as a core pathological event in kidney disease

**FIGURE 2:**
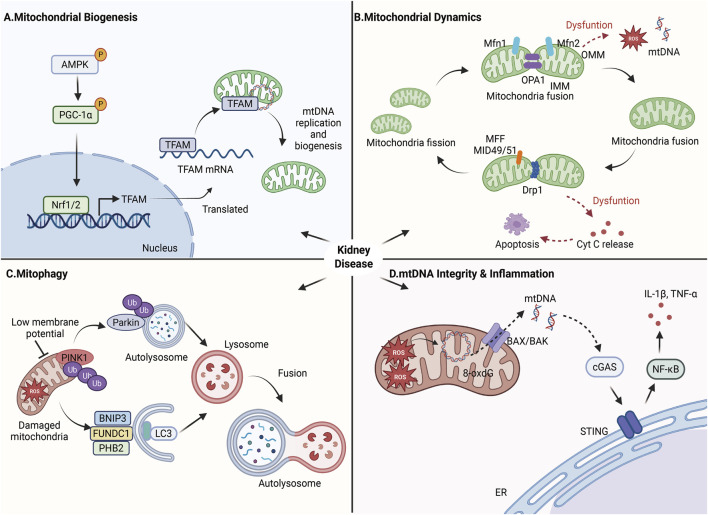
Mitochondrial quality control pathways involved in kidney disease. **(A)** The AMPK–PGC-1α–Nrf1/2–TFAM axis promotes mtDNA replication and mitochondrial biogenesis. **(B)** Imbalanced mitochondrial fusion (Mfn1/2, OPA1) and fission (Drp1, MFF, MID49/51) leads to ROS overproduction, mtDNA damage and cytochrome-c–mediated apoptosis. **(C)** PINK1/Parkin- and receptor-mediated mitophagy (BNIP3, FUNDC1, PHB2) remove damaged mitochondria via autolysosomes. **(D)** Oxidative mtDNA lesions and BAX/BAK-dependent mtDNA release activate the cGAS–STING–NF-κB pathway and pro-inflammatory cytokines, driving kidney injury.

CKD encompasses a wide range of etiologies, with significant differences in its pathophysiological drivers. Mitochondrial dysfunction constitutes a common pathogenic nexus across diverse kidney diseases. The AKI-to-CKD transition model captures acute mitochondrial damage with the potential for recovery, whereas established CKD is characterized by irreversible organelle loss and epigenetic reprogramming, which may alter treatment responsiveness. Mitochondrial biogenesis, dynamics (fusion/fission), and mitophagy, together with the genomic stability of nDNA and mtDNA, collectively maintain mitochondrial abundance, structural integrity, and bioenergetic competence. Figure 2 summarizes key components of mitochondrial quality control and their pathological roles in kidney disease.

Mitochondrial biogenesis is coordinated by nuclear and mitochondrial genomes to replenish mitochondrial mass and restore function. This program is governed primarily by peroxisome proliferator-activated receptor gamma coactivator 1α (PGC-1α), which cooperates with multiple transcription factors to regulate mitochondrial gene expression (Li P. A. et al., 2017; Scarpulla et al., 2012). PGC-1α translocates into the nucleus, where it interacts with nuclear respiratory factor 1/2 (Nrf1/Nrf2) to activate mitochondrial transcription factor A (TFAM), in turn facilitating mtDNA replication, transcription, and the biogenesis of new mitochondria (Li P. A. et al., 2017; Uittenbogaard and Chiaramello, 2014). Experimental evidence indicates that PGC-1α overexpression improves mitochondrial function and renal recovery after acute kidney injury (AKI), in part by alleviating endoplasmic reticulum stress through the unfolded protein response (UPR) and reducing mitochondria-/ER-associated apoptosis (Pan et al., 2022). PGC-1α is highly expressed in proximal tubules to meet the energetic demands of fatty acid β-oxidation; after oxidative injury, PGC-1α overexpression can restore mitochondrial and cellular function, underscoring the reparative role of biogenesis in tubular damage (Rasbach and Schnellmann, 2007). Podocytes exhibit lower PGC-1α expression levels. Moderate activation maintains mitochondrial homeostasis, whereas excessive activation leads to collapsing glomerulopathy (Li S. Y. et al., 2017). In the db/db model, reduced pyruvate kinase M2 (PKM2) activity promotes tubular cell death and fibrosis via HIF-1α–mediated suppression of PGC-1α, coupled with glycolytic reprogramming and mitochondrial dysfunction (Park et al., 2025). Renal mitochondrial biogenesis is also governed by energy-sensing pathways including mechanistic target of rapamycin (mTOR) and AMP-activated protein kinase (AMPK). AMPK enhances PGC-1α activity through phosphorylation (Thr177 and Ser539), thereby promoting biogenesis (Jäger et al., 2007), whereas mTORC1 deficiency reduces PGC-1α expression and mitochondrial abundance (Grahammer et al., 2014). Collectively, dysregulated mitochondrial biogenesis contributes to the pathogenesis of AKI, diabetic kidney disease (DKD), and other renal disorders.

Mitochondrial dynamics maintain organelle morphology and network distribution through coordinated fission and fusion, largely mediated by dynamin-family GTPases. During fission, phosphorylated dynamin-related protein 1 (DRP1) translocates to the outer mitochondrial membrane (OMM) and interacts with receptors including mitochondrial fission factor (MFF) and MID49/MID51 to drive mitochondrial division and segregate damaged segments (Tilokani et al., 2018). DRP1 upregulation has been linked to mitochondrial fragmentation, impaired ATP generation, and reduced respiratory-chain activity, thereby exacerbating cyst growth and renal dysfunction in autosomal dominant polycystic kidney disease (Cassina et al., 2020). Knockout of Drp1 in podocytes blocks excessive mitochondrial fission, improves mitochondrial adaptability, and prevents progression of DKD in mice (Ayanga et al., 2016). Excessive fission also facilitates cytochrome c release and apoptosis, whereas fission inhibition can restrain cell death. Conversely, impaired fission may lead to excessive fusion and accumulation of enlarged megamitochondria. Fusion is mediated by mitofusin 1/2 (MFN1/2) on the OMM and optic atrophy 1 (OPA1) on the inner mitochondrial membrane (IMM) (Liu Y. T. et al., 2024). Fusion defects lead to mitochondrial fragmentation and uneven energy supply within podocytes, causing podopodia to fuse and detach easily. This enlarges the pore size of the filtration membrane, triggering proteinuria and potentially progressing to focal segmental glomerulosclerosis (FSGS) over time. Research indicates that Mfn2 overexpression mitigates podocyte injury induced by puromycin aminonucleoside (PAN), while Mfn2 downregulation impairs the renal protective activity of astragaloside IV (AS-IV), likely via its regulatory impact on mitophagy (Yuan et al., 2025). OPA1 is enriched at cristae; its deficiency disrupts cristae ultrastructure, reduces ATP production, and increases ROS generation (Gall et al., 2015). Excess ROS not only damages DNA but also amplifies inflammatory signaling, accelerating renal inflammation and fibrosis. Endothelial Opa1 deletion alters vascular reactivity and heightens oxidative stress in aged kidneys (Turnaturi et al., 2025). Empagliflozin has been reported to upregulate OPA1 via AMPK signaling, enhance mitochondrial fusion, reduce renal inflammation, and alleviate ischemia–reperfusion injury (IRI) (Yang et al., 2023). OPA1 may be a crucial factor in protecting vascular health in target organs such as the kidneys. Thus, targeting mitochondrial dynamics represents a promising therapeutic avenue in kidney disease.

Mitophagy selectively recognizes and degrades dysfunctional or senescent mitochondria, thereby preserving mitochondrial quality and cellular homeostasis (Onishi et al., 2021). PINK1/Parkin-dependent mitophagy has been shown to attenuate tubular epithelial apoptosis and tissue injury in AKI, partly by limiting mtROS and restraining NLRP3 inflammasome activation (Lin et al., 2019). Tang et al. observed that Pink1 or parkin knockout exacerbated IRI-induced oxidative stress, inflammation, and cell death, accompanied by increased accumulation of damaged mitochondria in proximal tubules (Tang et al., 2018). However, cells also possess a parkin-independent mitochondrial autophagy pathway, primarily mediated directly by autophagy receptor proteins on the outer mitochondrial membrane (OMM). BCL2/adenovirus E1B 19-kDa interacting protein 3 (BNIP3), for example, mediates hypoxia-associated mitophagy; in hypoxia/reoxygenation (H/R)-treated proximal tubule cells, BNIP3 expression increases, and BNIP3 overexpression reverses the suppression of H/R-induced mitophagy caused by HIF-1α deficiency (Fu et al., 2020). Deficiency of Pink1, Park2, or Bnip3 worsens IRI by enhancing mitochondrial damage, ROS production, tubular apoptosis, and tubulointerstitial inflammation (Tang et al., 2018; Tang et al., 2019). FUNDC1 also functions as a mitophagy receptor and links mitophagy with biogenesis, helping maintain mitochondrial quality control via the PGC-1α/NRF1 axis (Liu L. et al., 2021). Prohibitin 2 (PHB2) has been implicated in mitophagy regulation, and N-acetylcysteine (NAC) has been reported to protect mitochondrial function via the Nrf2/PHB2 pathway in β cells (Liu S. et al., 2024). Together, ubiquitin-dependent and receptor-mediated mitophagy pathways are integral to mitochondrial homeostasis and represent potential targets for renal therapy.

Mitochondrial structure and function depend on the coordinated expression of proteins encoded by nDNA and mtDNA, and mtDNA copy number is often used as a surrogate indicator of mitochondrial status (Sena and Chandel, 2012). In a risk-modeling study of 2,943 CKD patients, mtDNA copy number bore a correlation with the progression of CKD and the risk of mortality (He et al., 2022). Because mtDNA is maternally inherited and particularly vulnerable to oxidative damage, the kidney owing to its high energy demand may accumulate pathogenic mtDNA mutations that impair respiratory-chain function, reduce ATP synthesis, and increase ROS production, thereby accelerating oxidative stress and cell death (Yan et al., 2013). mtDNA integrity also intersects with biogenesis, dynamics, and quality control. In a cisplatin-induced AKI model, aspirin mitigated mtDNA damage by activating the AMPK–PGC-1α axis and upregulating biogenesis-related genes (PGC-1α, NRF1, TFAM) (Tong et al., 2023). Research indicates that Drp1 overexpression provokes mitochondrial dysfunction and cytoplasmic mtDNA stress, thereby activating the cGAS-STING pathway, triggering autophagy, and facilitating the progression of esophageal squamous cell carcinoma (ESCC) (Li et al., 2022). Methylene blue has been reported to facilitate mtDNA repair via activation of Nrf2/ARE signaling and engagement of base excision repair (BER) mechanisms in cisplatin injury (Samoylova et al., 2023). Increasing evidence indicates that multiple mtDNA repair pathways, including BER and double-strand break repair, are mobilized during renal injury and recovery, underscoring the central importance of mitochondrial genome maintenance in kidney disease.

### 3.3 Renal mitochondrial damage exerts feedback effect on the gut

Renal mitochondrial injury is not confined to the kidneys; it can also reciprocally impair intestinal barrier integrity and motility via the kidney–gut axis. Nishiyama et al. observed in a 5/6 nephrectomy CKD mouse model that renal dysfunction was accompanied by gut microbiota dysbiosis and colonic inflammation, with dysbiosis being a key driver of intestinal motility disorders (Wang et al., 2026). Following broad-spectrum antibiotic treatment to eradicate the microbiota, colonic inflammation and motility abnormalities resolved synchronously, directly demonstrating that kidney injury-induced intestinal pathology is microbiota-mediated (Nishiyama et al., 2019). Huang et al. demonstrated that protein-bound uremic toxin indole-3-carbinol sulfate (IS), which accumulates in CKD, activates the intestinal epithelial AhR-IRF1 pathway, inhibits transcription of the mitochondrial disorganization protein DRP1, thereby blocking the mitophagy flux. This leads to mitochondrial damage, disruption of tight junctions, and impaired barrier function in IECs (Huang et al., 2020). Since DRP1 deficiency alone causes accumulation of autophagy intermediates like P62 and LC3-II, kidney-derived IS exacerbates intestinal mitochondrial dysfunction via the IRF1-DRP1 axis (Yamada et al., 2018). In a 5/6 nephrectomy CKD model, IS accumulation directly inhibits mitochondrial autophagy in IECs, reduces tight junction protein expression, and induces apoptosis, leading to intestinal barrier damage (Zheng et al., 2024). Under uremic conditions, endothelial cells produce DAMPs and amplify inflammatory signals via the TLR4/NF-κB pathway (Martin-Rodriguez et al., 2015), while the intestinal barrier becomes more permeable due to disrupted urea metabolism and damage to tight junctions (Vaziri et al., 2013). During ischemia-reperfusion injury, renal tubular epithelial cells undergo a burst of mtROS production, which weakens TFAM’s protective capacity over mtDNA, leading to the release of oxidized mtDNA into the circulation. Circulating oxidized mtDNA can be regarded as a specific type of DAMPs, which may be recognized by TLR9 on intestinal epithelial cells, activating local NLRP3 inflammasomes and inducing mitochondrial dysfunction (Xie et al., 2017). Based on clinical observations of ESRD patients, Durand et al. proposed that hydrogen sulfide accumulated in the kidneys can regulate mitochondrial energy production and redox balance, alter systemic energy metabolism, and affect intestinal mitochondrial homeostasis (Durand et al., 2018). In chronic kidney disease, there is a close association between mitochondrial dysfunction and gut microbiota dysbiosis. PGC-1α, a key regulator of mitochondrial biogenesis, electron transport chain assembly, and mitochondrial DNA replication, is significantly downregulated in CKD; this leads to impaired mitochondrial biogenesis, defects in oxidative phosphorylation, and abnormal organelle dynamics in intestinal mucosal tissues (Su et al., 2017; Eirin et al., 2017). Collectively, these findings indicate that renal mitochondrial dysfunction can amplify intestinal injury through a uremic toxin–microbiota–mitochondria cascade, highlighting renal mitochondrial damage as a potential therapeutic target for gastrointestinal complications in CKD.

## 4 Mitochondria-mediated gut-kidney axis interactions: key signaling pathways and molecular mechanisms

### 4.1 Microbiota metabolites-mitochondrial receptor axis

Metabolites derived from gut microbiota regulate energy homeostasis, oxidative stress, and inflammatory responses in renal parenchymal cells by directly acting on mitochondria or activating membrane receptors, thereby influencing renal physiological and pathological processes. Consequently, they serve as key signaling molecules in bidirectional gut-kidney axis communication (Rysz et al., 2021). Dysbiosis of the gut microbiota is associated not only with the cessation of beneficial metabolite production such as short-chain fatty acids and IPA, but also with increased generation of harmful metabolites and uremic toxins.

SCFAs are the primary products of dietary fiber fermentation by gut microbiota. In healthy individuals, SCFAs are detected via G protein-coupled receptors (GPCRs) such as GPR41 (Free Fatty Acid Receptor 3, FFAR3), GPR43 (Free Fatty Acid Receptor 4, FFAR4), GPR109 A (hydroxycarboxylic acid receptor 2, HCAR2), and olfactory receptor 78 (Olfr78). Additionally, SCFAs can act as histone deacetylase (HDAC) inhibitors. SCFAs can be detected by at least four distinct GPCRs in host tissues, with these receptors distributed in immune cells and the portal venous system, among other locations. Among these, FFAR2 (GPR43) and FFAR3 (GPR41) are activated by the three major SCFAs: acetate, butyrate, and propionate. GPR109 A is the specific receptor for butyrate, while OLFR78 is the specific receptor for acetate and propionate (Pluznick, 2016). Short-chain fatty acids in the blood are absorbed by the kidneys via G protein-coupled receptors (GPCRs) or MCTs. Studies have shown that GPR41, GPR43, and Olfr78 are expressed throughout the kidney and renal arteries (Pluznick et al., 2013). Studies indicate that in renal tubular epithelial cells and vascular smooth muscle cells (VSMCs), GPR41/43 translocates to the outer mitochondrial membrane (OMM), directly interacts with the mitochondrial fusion protein Mfn2, regulates mitochondrial membrane potential (ΔΨm), and inhibits ROS production (Pluznick et al., 2013). Building upon the understanding that short-chain fatty acids regulate renal mitochondrial homeostasis through multiple receptors, elucidating the mechanistic differences among various SCFAs is essential.

Butyrate is the most extensively studied metabolite among SCFAs. In human glomerular microvascular endothelial cells (hgMVECs), butyrate increases mitochondrial mass and upregulates the mitochondrial biogenesis regulator PGC-1α, while attenuating the LPS-induced rise in maximal mitochondrial respiratory capacity, thereby alleviating metabolic stress associated with mitochondrial hyperactivation (Nicese et al., 2023). Butyrate can reduce endothelial cell proliferation, likely due to its ability to inhibit oxidative stress through GPR41/43 activation, decrease ROS production, and restore nitric oxide (NO) generation (Robles-Vera et al., 2020). In DKD mouse models, butyrate also promotes mitochondrial biogenesis via GPR109A, increases ATP production, alleviates high-glucose-induced mitochondrial swelling and cristae disruption, thereby protecting the glomerular filtration barrier (Felizardo et al., 2019). As an HDAC inhibitor, butyrate can traverse the mitochondrial outer membrane and inhibit mitochondrial matrix HDAC1/2, increasing mitochondrial protein acetylation and enhancing electron transport chain (ETC) complex I and III activity (Kumar et al., 2017). Clinically, CKD patients exhibit reduced SCFA levels, suggesting that supplementation with butyrate could contribute to retarding the progression of CKD (Wang et al., 2019). Cai K et al. found reduced serum butyrate levels in the DKD group (Cai et al., 2022). These findings indicate that butyrate improves mitochondrial biogenesis in glomerular endothelial cells via receptor-mediated signaling while restraining hyperproliferation and oxidative stress, highlighting a potential intervention target to correct SCFA deficiency and delay renal injury.

In CKD rat models, propionic acid supplementation activates GPR43 in renal tissue, promoting mitophagy via the AMPK/mTOR signaling pathway to clear damaged mitochondria and reduce inflammation triggered by mitochondrial DNA release (He et al., 2024). Olfr78 is highly expressed in the mitochondrial-rich region of the juxtaglomerular apparatus (JGA). Upon propionic acid activation of Olfr78, mitochondrial pyruvate dehydrogenase (PDH) phosphorylation is enhanced, increasing ATP production and stimulating renin release (Xu and Pluznick, 2022). Additionally, propionic acid modulates sympathetic nervous system activity via GPR41, indirectly improving renal mitochondrial energy metabolism and reducing oxidative stress levels (Pluznick et al., 2013). However, acetate exhibits relatively weaker regulatory effects on renal mitochondria. In hgMVECs, acetate treatment did not significantly alter mitochondrial mass or respiratory function, suggesting receptor-specific differences in interactions between SCFAs and GPCRs (Nicese et al., 2023). Collectively, acetate, propionate, and butyrate are microbiota-derived metabolites with mitochondria-protective properties spanning mitochondrial energy metabolism, biogenesis, and antioxidant defenses, thereby conferring renoprotective effects.

The indole and its derivatives produced by gut microbiota through tryptophan metabolism exert concentration-dependent effects on mitochondria. At physiological concentrations, indole-3-propionic acid (IPA) enters mitochondria and directly scavenges hydroxyl radicals (·OH), thereby reducing ROS damage to mitochondrial cristae and mtDNA. Studies indicate that indole-3-propionic acid (IPA) levels are significantly reduced in the serum of DKD patients, positively correlated with estimated glomerular filtration rate (eGFR), and negatively correlated with the urine albumin-to-creatinine ratio (UACR). IPA activates the SIRT1/PGC-1α mitochondrial protection pathway by inhibiting the phosphorylation-mediated ubiquitination degradation of silent information regulator 1 (SIRT1), thereby improving renal injury (Zeng et al., 2025). Research has demonstrated that IPA is considered an ideal potent antioxidant capable of effectively promoting the repair of mitochondrial structural and functional damage. It serves as a crucial biomarker and nephroprotective agent for preventing the progression of CKD (Dragicevic et al., 2011). When gut microbiota imbalance or declining renal function occurs, indole is converted into indole sulfate (IS) in the liver. Circulating through the bloodstream, IS enters the mitochondria of organs such as the kidneys and heart, where it exerts toxic effects. As a prototypical entero-derived uremic toxin, IS inhibits mitochondrial organic anion transporters (OAT1/OAT3), reducing antioxidant uptake into the mitochondrial matrix. This increases mtROS production and induces mitochondrial apoptosis in renal tubular epithelial cells (Vanholder et al., 2014). Reducing IS accumulation may represent a promising therapeutic strategy for alleviating CKD-associated intestinal dysfunction. Other uremic toxins such as pCS and TMAO, derived directly or indirectly from gut microbiota metabolism, are also closely implicated in renal disease.

TMAO is a product of gut microbiota metabolism of choline and L-carnitine, exhibiting significantly elevated levels in CKD/DKD patients. It exacerbates renal injury by activating mitochondrial-associated inflammatory pathways. TMAO triggers mtROS production by binding to Toll-like receptor 4 (TLR4) on the mitochondrial membrane. In a rat model of diabetic nephropathy, TMAO treatment markedly increased mtROS levels in renal tissue, activated NLRP3 inflammasomes, promoted IL-1β and IL-18 release, and subsequently induced abnormal mitochondrial autophagy and fibrosis in tubular epithelial cells (Wang et al., 2020). The TMAO-TLR4 interaction activates the NF-κB pathway, downregulates key mitochondrial autophagy genes PINK1 and Parkin, leading to accumulation of damaged mitochondria and release of mitochondrial DNA (mtDNA), thereby amplifying the inflammatory response (Tang et al., 2015). Clinical studies confirm that plasma TMAO levels in CKD patients negatively correlate with renal mitochondrial mtDNA copy number and mitochondrial respiratory chain enzyme activity, serving as an independent risk factor for predicting kidney disease progression (Missailidis et al., 2016). Thus, regulating the gut microbiota emerges as a potential intervention strategy to delay uremia progression.

Table 2 summarizes how gut microbial metabolites act on the kidneys through mitochondria. In summary, the microbiota-metabolite-mitochondrial receptor axis constitutes a core signaling pathway for gut-kidney axis interactions through key receptors such as GPCRs, SIRT1, and TLR4. In the pathological state of CKD/DKD, reduced production of protective metabolites like SCFAs and IPA, or inhibition of their receptor pathways, combined with accumulation of pathogenic metabolites such as TMAO and PCS or activation of their inflammatory pathways, collectively leads to mitochondrial dysfunction and drives the progression of renal injury. Intervention strategies targeting gut microbiota metabolites and key mitochondrial receptors hold promise as a novel therapeutic direction for kidney diseases.

**TABLE 2:** Summary of the gut microbiota metabolites-mitochondria-kidney axis.

Study type	Microbial metabolite	Key receptors/ Targets	Mitochondrial mechanisms	References
*In vitro*	Butyrate	PGC-1α	Upregulates PGC-1α expression; attenuates LPS-induced increases in maximal mitochondrial respiration	Nicese et al. (2023)
​	Butyrate	GPR109 A	Promotes mitochondrial biogenesis, increases ATP production, and ameliorates mitochondrial damage	Felizardo et al. (2019)
​	Indoxyl sulfate (IS)	OAT1, OAT3	Inhibits OAT1/OAT3, leading to increased mtROS production	Vanholder et al. (2014)
​	TMAO	TLR4	Activates the NF-κB pathway, inhibits mitophagy, resulting in accumulation of damaged mitochondria and release of mtDNA	Tang et al. (2015)
Animal Studies	Butyrate	HDAC1/2	Inhibits matrix HDAC1/2, enhances mitochondrial protein acetylation, and increases, ETC., complex I and III activity	Kumar et al. (2017)
​	Propionate	GPR43/FFAR2	Activates the AMPK/mTOR pathway, promotes mitophagy, and reduces mitochondrial DNA release	He et al. (2024)
​	Propionate	GPR41/FFAR3	Modulates sympathetic nervous system activity, improves renal mitochondrial energy metabolism, and reduces oxidative stress	Pluznick et al. (2013)
​	Indole-3-propionic acid (IPA)	SIRT1/ PGC-1α	Upregulates PGC-1α, promotes mitochondrial biogenesis, and alleviates renal injury	Zeng et al. (2025)

### 4.2 Inflammation-oxidative stress amplification axis

Mitochondria amplify inflammation and oxidative-stress signaling between the gut and kidneys by coordinating ROS generation and antioxidant defenses. When gut microbiota imbalance occurs, bacterial-derived uremic toxins such as TMAO, LPS, accumulate in the bloodstream. These toxins can enter renal tubular epithelial cells via organic anion transporters, directly inhibiting mitochondrial respiratory chain complexes I and III. This process contributes to glomerular and tubular injury while promoting inflammation and oxidative stress, thereby exerting pathogenic effects (Gryp et al., 2020). Research indicates that TMAO enters renal tubular epithelial cells via OAT1/3, inhibiting respiratory chain complexes I and III. This leads to increased electron leakage and the generation of substantial ROS (Taguchi et al., 2021). Excessive ROS damages mtDNA, generating oxidative adducts such as eight-oxodeoxyguanosine (8-oxoG), which disrupt mitochondrial transcription, impair ATP synthesis, and further intensify oxidative stress (Pizzino et al., 2017). Damaged mitochondria subsequently release mtDNA fragments into the cytosol, where they are sensed by cyclic GMP–AMP synthase (cGAS), triggering activation of the cGAS–STING–NF-κB cascade and inducing pro-inflammatory cytokine transcription, thereby promoting systemic inflammation (Lee et al., 2024). TMAO exacerbates oxidative stress by upregulating NOX4 and downregulating SOD, increasing ROS and peroxynitrite production, inducing endothelial dysfunction and vascular inflammation, and worsening tubulointerstitial hypoxia and fibrosis (Lim et al., 2021). Excess mtROS can also activate NF-κB and the NLRP3 inflammasome, downregulate tight junction proteins, and increase intestinal permeability (Guerbette et al., 2025). Zhao et al. observed in an IRI-AKI mouse model that elevated mtROS in renal tubular epithelial cells promotes TFAM degradation and mtDNA release into the cytoplasm. Cytoplasmic mtDNA synergistically activates the cGAS-STING pathway with LPS, exacerbating renal inflammation (Zhao et al., 2021). Circulating LPS and mtDNA, both acting as DAMPs, can engage TLR4 on renal tubular cells, inducing MyD88-dependent NOX2 assembly, further amplifying ROS bursts and suppressing PGC-1α–mediated mitochondrial biogenesis (Tao et al., 2025). Thus, the gut-kidney axis initiates a positive feedback loop through mtROS, involving intestinal barrier disruption, microbiota translocation, and renal mtDNA leakage, which continuously amplifies inflammatory and oxidative stress responses.

Under physiological conditions, SCFAs produced by gut microbiota fermentation of dietary fiber act as negative regulators suppressing inflammation and oxidative stress (Nicese et al., 2023). In CKD states, the abundance of butyrate-producing bacteria significantly declines, leading to reduced SCFA production. This failure to activate the Nrf2-ARE antioxidant pathway results in mtROS accumulation, further exacerbating oxidative stress (Wang et al., 2019). The fucoidan intervention enriched SCFA-producing bacteria, increased cecal acetate, elevated renal ATP, and ameliorated mitochondrial dysfunction, while attenuating renal inflammation and fibrosis through MAPK inhibition (Zhong et al., 2024). These studies suggest that SCFA could serve as a critical determinant of exacerbated inflammation-oxidative stress responses. Aging and metabolic status may further modulate this axis. The aged rats display diminished complex I activity alongside enhanced mitochondrial fragmentation, and produce higher levels of ROS in response to TMAO stimulation, implying that age-associated declines in mitochondrial plasticity intensify susceptibility to oxidative and inflammatory injury (Nesterova et al., 2024). Overall, within gut–kidney crosstalk, microbial metabolites act as initiating signals that provoke mtROS overproduction, activate NF-κB/NLRP3 pathways, and promote inflammatory mediator release. Concurrent mtDNA damage and mitochondrial membrane potential loss reinforce feed-forward loops that perpetuate oxidative stress. Therapeutic strategies that reduce the systemic burden of deleterious microbial metabolites, blunt ROS surges, strengthen mitochondrial antioxidant capacity, and enhance mitochondrial biogenesis may enable multi-target, stage-adapted, and precision interventions for CKD.

### 4.3 Iron death regulatory mechanisms

Beyond their role in energy metabolism, mitochondria participate in regulating ferroptosis by governing iron handling, lipid peroxidation, ROS generation, and antioxidant homeostasis. Mitochondria influence ferroptotic signaling in part through iron homeostasis. Mitochondria initiate ferroptosis signaling through regulating iron homeostasis. Mitochondrial ferritin (MtFt) sequesters labile iron and can suppress ferroptosis; however, under cellular stress, Fe^2+^ import mediated by mitoferrin-1/2 (MFRN1/2) may exceed the matrix capacity for utilization and storage, leading to Fe^2+^ accumulation and triggering a burst of lipid ROS (Wu and Chen, 2022); MtFt deficiency expands the mitochondrial labile iron pool (LIP), reduces glutathione peroxidase 4 (GPX4) activity, and accelerates ferroptosis onset (Wang et al., 2022). In addition, the mitochondrial respiratory chain represents a major ROS source that fuels ferroptosis. Superoxide generated at complex III can be converted to hydrogen peroxide (H_2_O_2_); in the presence of free iron, the Fenton reaction produces hydroxyl radicals (·OH) that directly attack polyunsaturated fatty acids (PUFAs) in mitochondrial membranes, initiating lipid peroxidation cascades central to ferroptotic execution (Lloyd et al., 1997). Mitochondrial glutathione reductase (GR) further maintains antioxidant capacity by sustaining glutathione (GSH) pools. When GR activity is impaired, GPX4 becomes functionally compromised, phospholipid hydroperoxides (PL-OOH) accumulate, and mitochondrial membrane potential collapses, culminating in ferroptosis (Wang et al., 2023).

Mitochondrial ferroptosis is not only an autonomous cellular process but also influences distal organ damage through metabolic and inflammatory signaling via the gut-kidney axis. In CKD, compromised intestinal barrier function facilitates endotoxin (LPS) translocation into the bloodstream. LPS activates TLR4/NF-κB signaling in renal macrophages, promoting mitochondrial NLRP3 inflammasome assembly. Inflammasome activation triggers caspase-1–mediated cleavage of gasdermin D (GSDMD), and the resulting membrane pores further intensify mitochondrial oxidative stress, promoting Fe^2+^ dysregulation and ferroptosis (Li et al., 2023). Clinical studies indicate that serum LPS levels in CKD patients positively correlate with mitochondrial LIP volume in the kidneys, and the expression of the ferroptosis marker 4-HNE is 3.1 times higher than in healthy individuals (Zhai et al., 2024). Research has revealed that LPS induces renal ferroptosis by enhancing mitochondrial oxidative stress and 4-HNE accumulation; activation of ALDH2 mitigates inflammation and oxidative stress via MAPK inhibition, thus enhancing renal function (Jin et al., 2023). Ding et al. demonstrated in a CCl_4_-induced renal injury model that fucoxanthin improves intestinal dysbiosis by reducing serum TMAO levels, upregulates the mitochondrial antioxidant pathway Nrf2/HO-1, restoring GPX4 levels, inhibiting mitochondrial iron overload and lipid peroxidation, suggesting a central role for mitochondrial function in ferroptosis regulation mediated by the gut-kidney axis (Ding et al., 2025). TMAO accumulation is also implicated as a driver of mitochondrial ferroptosis in CKD. Mechanistically, TMAO activates ZBP1-mediated PANoptosis signaling and promotes assembly of inflammasome-related complexes (ASC, RIPK3, and caspase-8). This complex reportedly enhances MFRN1/2 localization at the inner mitochondrial membrane via Ser256 phosphorylation, increasing Fe^2+^ import and exacerbating lipid peroxidation cascades (Wang et al., 2025). In contrast, SCFAs produced by beneficial microbes exert protective effects, partly through mitochondria-related signaling pathways. Kidney tea extract reduces proteinuria and improves glomerular filtration by increasing cecal butyrate, inhibiting ferroptosis in tubular epithelial cells, and restoring mitochondrial membrane potential, thereby delaying type 2 diabetes–associated diabetic nephropathy progression (Zhou et al., 2024). Sodium butyrate reduces mitochondrial iron load by upregulating nuclear coactivator 4 (NCOA4) expression in proximal tubular epithelial cells, promoting its interaction with ferritin heavy chain (FTH1), and accelerating ferritin degradation via autophagy, thereby significantly decreasing renal ferroptosis incidence in a colorectal cancer liver metastasis mouse model (Liu et al., 2025). Clinically, fecal SCFA concentrations are significantly reduced in CKD and correlate positively with estimated glomerular filtration rate (eGFR), suggesting that SCFA deficiency may serve as a potential biomarker for CKD progression (Obeid et al., 2024). In addition, the gut microbial metabolite 3-hydroxyphenylacetic acid has been reported to preserve mitochondrial function by upregulating GPX4, thereby inhibiting ferroptosis and attenuating renal injury (Mao et al., 2024). Together, these findings indicate that gut microbiota–derived metabolites exert bidirectional control over renal ferroptosis, with outcomes determined by metabolite composition and overall microbial community balance. Mitochondria orchestrate ferroptosis through regulation of iron metabolism, initiation of lipid peroxidation, and maintenance of antioxidant defenses. Mitochondrial dysfunction driven by metabolic and inflammatory signals along the gut–kidney axis therefore constitutes a critical bridge linking intestinal homeostasis to renal injury. Therapeutic strategies aimed at restoring mitochondrial function while reprogramming gut–kidney axis signaling may provide novel avenues for intervention in ferroptosis-associated diseases.

Beneficial metabolites such as butyrate, propionate, and IPA activate GPR41/43, GPR109A, and SIRT1-PGC-1α to promote biogenesis and protect mitochondrial function. Pathogenic metabolites trigger downstream events of mitochondrial dysfunction by binding to receptors such as AhR, TLR4, and OATs. As upstream mechanisms, gut metabolites influence central mitochondrial effectors; mitochondrial dysfunction then converts metabolic stress into downstream responses such as the release of inflammasomes, excessive ROS production, and iron pool dysregulation. Microbiota metabolites further influence mitochondrial oxidative stress and iron metabolism by regulating mitochondrial biogenesis, dynamics, and autophagy, ultimately determining the mode of cell death in intestinal and renal cells and the extent of organ damage. Together, these three components form the regulatory core of the mitochondria-gut-kidney axis.

**FIGURE 3:**
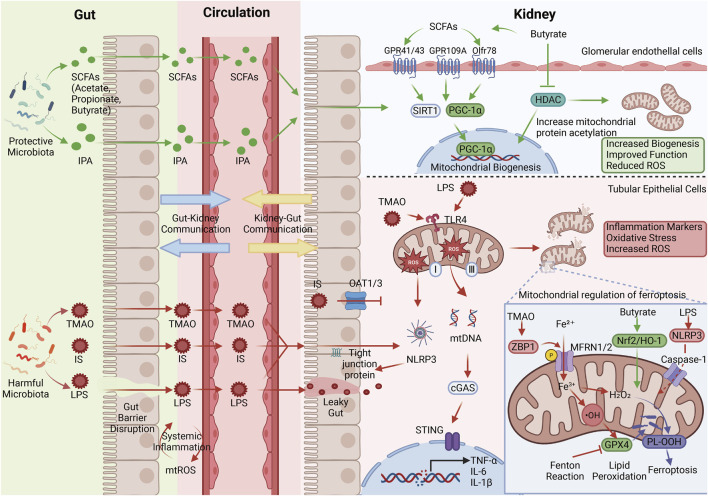
Mitochondria-mediated interaction mechanism of the gut-kidney axis. SCFAs and IPA promote mitochondrial biogenesis, improve function, and reduce ROS by activating SIRT1–PGC-1α or inhibiting HDAC. TMAO, IS, and LPS exacerbate mitochondrial dysfunction and renal injury by inducing oxidative stress, inflammatory pathways, and ferroptosis.

As illustrated in Figure 3, beneficial metabolites SCFAs and IPA enter the circulatory system and act on receptors such as GPR41/43, GPR109A, and Olfr78 to activate the SIRT1-PGC-1α signaling axis, consequently ameliorating mitochondrial biogenesis. Alternatively, they enhance mitochondrial protein acetylation levels by inhibiting HDACs, improving mitochondrial function and reducing ROS production. Conversely, dysbiosis produces uremic toxins like TMAO, IS, and LPS, disrupting the intestinal barrier and activating the TLR4-mtROS axis. This directly damages renal mitochondrial function through oxidative stress and mtDNA release. Harmful metabolites like TMAO and LPS amplify renal inflammation and tissue injury by inducing iron homeostasis disruption, lipid peroxidation, and promoting ferroptosis through GPX4 inhibition. Conversely, butyrate counteracts oxidative stress via the Nrf2/HO-1 pathway, inhibiting ferroptosis progression. Microbial metabolites mediate interactions between mitochondria and the kidney, making mitochondrial function a key focus in regulating gut-kidney interactions.

## 5 Targeting mitochondrial regulation of the gut-kidney axis: novel therapeutic strategies for disease management

### 5.1 Dietary intervention

Dietary interventions have arisen as a pivotal therapeutic strategy for controlling CKD and its complications. These interventions exert their effects by modulating gut microbiota composition, enhancing intestinal barrier integrity, regulating microbial metabolites, and restoring mitochondrial function (Kalantar-Zadeh et al., 2020). Recent studies have demonstrated that specific dietary regimens, such as fiber-rich intake, ketogenic diets, and low-protein plant-based diets, offer promising therapeutic avenues for kidney disease (Mafra et al., 2021). However, the mechanisms underlying dietary intervention’s effects on gut metabolites and renal health remain unclear. From a mitochondrial perspective, we demonstrate that gut metabolites can influence kidney health by affecting mitochondrial function.

A high-fiber diet significantly enriches SCFA-producing bacteria, such as *Bifidobacterium* and *Akkermansia*. Mechanistically, it delays the progression of diabetic nephropathy by activating GPR43 and GPR109 A receptors in renal tubular epithelial cells, thereby attenuating mitochondrial ROS production (Li et al., 2020). Furthermore, high-fiber intake promotes acetate production, which activates intestinal GPR43 to repair the gut barrier, while simultaneously suppressing renal sympathetic nerve activity to improve sodium excretion (Marques et al., 2017). Butyrate confers protection against AKI by inhibiting HDACs, thereby reducing tubular epithelial cell apoptosis, inflammation, and oxidative stress (Liu Y. et al., 2021). Clinical evidence confirms a strong correlation between high-fiber diets and reduced inflammation and improved prognosis in CKD patients, effects likely mediated by SCFA-driven systemic anti-inflammatory mechanisms and gut barrier reinforcement (Krishnamurthy et al., 2012). High-fiber diets have emerged as a key strategy for maintaining gut-kidney axis homeostasis.

The ketogenic diet (KD), characterized by high fat, low carbohydrate, and moderate protein intake, remodels the gut microbiota by altering the host metabolic state (Ma et al., 2018). In DN mice, KD has been shown to reverse mesangial matrix expansion and restore glomerular filtration rates (Poplawski et al., 2011) It also alleviates oxidative stress by elevating β-hydroxybutyrate (βOHB), which inhibits HDAC activity and activates the NRF2 antioxidant pathway (Wan et al., 2023). However, the renal effects of KD are not universally protective. Jia et al. reported that in spontaneously hypertensive rats (SHR), KD-induced metabolic dysregulation and the inhibition of renal mitochondrial autophagy exacerbated renal dysfunction (Jia et al., 2021). Individuals with a dietary fiber intake of >25 g/day had a 37% lower all-cause mortality rate and a 29% lower C-reactive protein level compared to those with an intake of <10 g/day, suggesting a systemic anti-inflammatory effect of a high-fiber diet. The mechanism may be related to the systemic anti-inflammatory effects mediated by short-chain fatty acids (SCFAs) and the protective role of the intestinal barrier (Joshi et al., 2024). This discrepancy suggests that the impact of KD on the gut-kidney axis is contingent upon the underlying disease status, renal stage, and dietary adherence, highlighting the need for personalized application.

In contrast to animal protein-dominant regimens, plant-based low-protein diets (PLADO) can reduce serum IS and pCS levels in patients with chronic kidney disease (CKD). This effect is associated with the restoration of the gut microbiota’s ability to produce butyrate, which helps repair the intestinal barrier, modulate the production of uremic toxins, slow the progression of chronic kidney disease, and reduce cardiovascular risk (Kalantar-Zadeh et al., 2020). In summary, dietary interventions represent a key therapeutic strategy targeting the microbiota-mitochondria-kidney axis by modulating microbial composition, metabolic activity, and proteomic functions to restore intestinal barrier integrity.

### 5.2 Probiotics

Probiotics have been demonstrated to exert anti-inflammatory and energy-regulatory effects via the gut-kidney axis. In a DN mouse model, a quadruple probiotic formulation enhanced renal cortical mitochondrial membrane potential, increased ATP production, and reversed hyperglycemia-induced intestinal barrier hyperpermeability and renal tubular energy depletion (Kuo et al., 2023). The single strain *Akkermansia muciniphila* (Akk) improves mitochondrial biogenesis by activating the renal cortical AMPK/PGC-1α axis, thereby reducing renal interstitial fibrosis and inhibiting renal inflammation (Pei et al., 2023). Similarly, *Lactobacillus acidophilus* KBL409 enhances mitochondrial OXPHOS efficiency in renal parenchymal cells, disrupting the oxidative stress-inflammation cycle (Park et al., 2024). Clinical studies indicate that oral administration of composite probiotics containing *Lactobacillus* or *Bifidobacterium* improves renal function in DN patients while elevating fecal butyrate and serum propionate levels (Han et al., 2021). Collectively, probiotics restore epithelial tight junctions, augment SCFA secretion, and modulate mitochondrial biogenesis, offering a safe, scalable approach to targeting the gut-kidney axis via mitochondrial pathways.

### 5.3 Fecal microbiota transplantation

FMT entails the systemic reconstruction of the intestinal microbiome by transferring intact microbiota from healthy donors to dysbiotic recipients. Recent studies indicate that a single FMT intervention can promote barrier repair, reduce the enteric accumulation of uremic toxins, and decelerate renal fibrosis (Liu et al., 2022). Elucidating the mechanism, Yu et al. showed in an ischemia-reperfusion injury (IRI) model that FMT increases colonic propionate concentrations and activates GPR43, which reduces mtROS production and preserves membrane potential, thereby improving renal function (Yu et al., 2025). Evidence from human clinical trials of FMT in CKD is extremely limited. A single-center, double-blind RCT study showed that oral FMT capsules can slow the progression of CKD and maintain stable renal function; however, this study had a small sample size, a short follow-up period, and did not use mitochondrial function or the intestinal barrier as direct endpoints (Arteaga-Muller et al., 2024).

Our review of interventions targeting the gut microbiota-mitochondria-kidney axis indicates that dietary modifications and probiotics represent non-pharmacological approaches with advantages including high safety and minimal side effects. FMT not only alleviates the uremic toxin burden but also provides a strategy for targeted therapy via donor-derived SCFAs. Continued research into these mechanisms will facilitate the development of precision therapeutics for kidney diseases.

## 6 Conclusion

CKD and its complications have become a pressing global public health challenge. Its pathogenesis involves a synergistic imbalance between the gut and kidney organs. Within this interactive network, mitochondria as the core organelles of cellular energy metabolism not only underpin the physiological functions of both the gut and kidneys but also serve as a pivotal mediator linking gut microbiota homeostasis to the progression of renal injury.

In the gut, mitochondria supply ATP via OXPHOS for IEC renewal, tight junction protein synthesis, and goblet cell secretion. They also sustain ISC properties through FAO and shape the microbial colonization microenvironment by regulating luminal oxygen concentration. Mitochondrial dysfunction increases intestinal epithelial cell apoptosis and reduces antimicrobial peptide secretion, triggering “leaky gut” and dysbiosis. In the kidney, mitochondria occupy 30% of the volume of renal tubular epithelial cells (RTECs). Their OXPHOS supports Na^+^-K^+^-ATPase-mediated reabsorption functions. Imbalances in mitochondrial biogenesis, dynamics, and autophagy directly cause mitochondrial swelling and cristae disruption in tubular mitochondria, accompanied by decreased activity of respiratory chain complex I/ IV activity and ROS accumulation, ultimately driving renal interstitial fibrosis and podocyte apoptosis. This represents a core pathological event in the progression from AKI to CKD.

Crucially, mitochondria mediate bidirectional interactions within the gut-kidney axis through multiple signaling pathways. First, gut microbial metabolites form a signaling bridge: beneficial metabolites like short-chain fatty acids regulate renal mitochondrial function via receptors such as GPR43, GPR109A, and Olfr78, while IPA directly scavenges mitochondrial ROS to protect mtDNA. Conversely, harmful metabolites like IS and TMAO produced by dysbiosis exacerbate renal mitochondrial damage by inhibiting the mitochondrial respiratory chain, blocking autophagy flux, and activating the NLRP3 inflammasome. Second, renal injury reciprocally impacts the gut. Uremic toxins accumulating in CKD suppress DRP1 expression in IECs, disrupting mitochondrial autophagy and causing intestinal barrier leakage. Meanwhile, gut-derived LPS and proinflammatory factors like TNF-α and IL-6 enter the kidneys via the circulatory system, further inhibiting the PGC-1α pathway in renal tubular mitochondria, thereby establishing a vicious cycle of gut dysbiosis - Mitochondrial dysfunction - a vicious cycle of intestinal-renal injury. Furthermore, mitochondria participate in ferroptosis by regulating iron metabolism and lipid peroxidation, emerging as a novel focus in gut-kidney axis research. Furthermore, mitochondria participate in ferroptosis by regulating iron metabolism and lipid peroxidation, emerging as a novel focus in gut-kidney axis research. This review establishes a complete cascade framework from microbial metabolite sensing and mitochondrial quality control imbalance to the development of kidney diseases, providing a theoretical basis for stage-specific interventions. We further elaborate that renal mitochondrial damage reversely disrupts intestinal homeostasis through the uremic toxin–microbiota–mitochondria feedback loop. Targeting mitochondrial function restoration thus offers a promising strategy for modulating this axis.

To address mitochondrial-mediated gut-kidney axis dysregulation, various clinical interventions have been developed, with non-pharmacological approaches being particularly prominent. High-fiber diets indirectly enhance renal mitochondrial OXPHOS efficiency by enriching SCFA-producing bacteria such as *Bifidobacterium* and *Akkermansia*. Specific probiotics like *Akkermansia muciniphila* and *Lactobacillus acidophilus* KBL409 not only repair the intestinal barrier but also activate the renal NRF2-SOD2 antioxidant pathway to protect mitochondria. FMT reduces renal mitochondrial damage by reconstituting healthy microbiota. In addition, emerging evidence indicates that probiotics including *Lactobacillus* and *Bifidobacterium* may attenuate systemic inflammation by reducing circulating levels of protein-bound uremic toxins such as indoxyl sulfate and p-cresyl sulfate, thereby offering a clinically translatable strategy for gut-kidney axis-targeted interventions (Chen et al., 2019). In addition, pharmacological strategies involving natural products that target mitochondrial oxidative stress pathways are gaining increasing experimental support. Gao et al. demonstrated that total saponins from Panax notoginseng can inhibit the phosphorylation of PI3K/AKT, thereby activating the nuclear translocation of NRF2, suppressing the activation of the TXNIP/NLRP3 inflammasome, and alleviating high-glucose-induced mitochondrial damage and oxidative stress in endothelial cells (Gao et al., 2025). Naringin significantly improves fluoroacetamide-induced cardiac and renal dysfunction by activating the NRF2 antioxidant pathway, enhancing the activity of systemic antioxidant enzymes such as GSH, SOD, and GPx, and reducing MDA and H_2_O_2_ levels (Ajibade et al., 2024). The above studies indicate that natural flavonoids regulate mitochondrial redox homeostasis through multiple targets, providing reliable evidence for a mitochondrial-targeted therapy for CKD. These interventions are characterized by high safety, cost-effectiveness, and suitability for long-term management, aligning with the chronic nature of CKD. They simultaneously improve overall metabolic indicators like blood glucose and lipids, achieving integrated gut-kidney therapy.

In summary, mitochondria play a dual role as both the energy engine and signaling hub within the gut-kidney axis: they serve as the energy source for physiological functions like intestinal epithelial renewal and renal reabsorption, act as a key converter for gut microbiota metabolite signals transmitted to the kidneys, and represent a core target for breaking the vicious cycle of gut-kidney injury. Several research gaps remain in this field: the interactions among mechanisms such as gut microbiota metabolites, mitochondrial homeostasis, and ferroptosis remain unclear; there is a lack of research on regulatory differences among CKD caused by different etiologies; clinical translational biomarkers and intervention targets have yet to be established; and there is still insufficient evidence of causality regarding the bidirectional gut-kidney interaction. Although the hypothesis that mitochondria serve as a hub for bidirectional regulation of the gut-kidney axis is biologically plausible across different etiologies of CKD, the current body of evidence is largely concentrated in models of diabetic nephropathy, ischemia-reperfusion injury, and acute kidney injury. Future research should prioritize etiology-stratified analyses, incorporate human cohorts with clearly defined primary diagnoses, and develop gut-mitochondrial axis biomarkers validated in diverse CKD populations. Deepening our understanding of the mitochondrial-mediated gut-kidney interaction mechanism and promoting the combined application of non-pharmacological and pharmacological interventions targeting mitochondria will provide novel pathways for the prevention and treatment of gut-kidney axis-related diseases such as CKD. This ultimately enables a therapeutic leap from symptomatic control to mechanism-based repair.

## Glossary

ATP: adenosine triphosphate
CKD: Chronic kidney disease
FMT: Fecal microbiota transplantation
nDNA: nuclear DNA
mtDNA: mitochondrial DNA
SCFAs: Short-chain fatty acids
TMAO: Trimethylamine N-oxide
IPA: Indole-3-propionic acid
LPS: Lipopolysaccharide
IS: Indoxyl sulfate
IECs: Intestinal epithelial cells
OXPHOS: Oxidative phosphorylation
ISC: Intestinal stem cell
FAO: Fatty acid β-oxidation
PC: Paneth cell
HFD: High-fat diet
Bax: Bcl-2 associated X protein
Bak: Bcl-2 antagonist/killer
ΔΨm: Mitochondrial membrane potential
UC: Ulcerative colitis
CTN: Citrinin
(HDAC: Histone deacetylase
H2S: Hydrogen sulfide
DAMPs: Damage-associated molecular patterns
Ahr: Aryl hydrocarbon receptor
PGC-1α: Peroxisome proliferator-activated receptor γ coactivator 1α
Nrf1/Nrf2: Nuclear respiratory factor 1/2
TFAM: Mitochondrial transcription factor A
UPR: Unfolded protein response
PKM2: Pyruvate kinase M2
mTOR: Mechanistic target of rapamycin
AMPK: AMP-activated protein kinase
DRP1: dynamin-related protein 1
OM: Mouter membrane
MFF: Mitochondrial fission factor
MFN1/2: Mitofusin 1/2
IM: Minner mitochondrial membrane
FSGS: Focal segmental glomerulosclerosis
PAN: Puromycin aminonucleoside
AS-IV: Astragaloside IV
OPA1: Optic atrophy 1
ROS: Reactive oxygen species
EC: Endothelial cells
IRI: Ischemia-reperfusion injury
PINK1: PTEN-induced kinase 1
E3 ubiquitin ligase: Parkin
BNIP3: Bcl-2 19-kDa interacting protein 3
H/R: Hypoxia/reoxygenation
BER: Base excision repair pathway
GPCR: G protein-coupled receptor
FFAR3: Free fatty acid receptor 3, G protein-coupled receptor 41
FFAR4: Free fatty acid receptor 4, G protein-coupled receptor 43
HCAR2: Hydroxycarboxylic acid receptor 2, G protein-coupled receptor 109A
Olfr78: Olfactory receptor 78
hgMVECs: Human glomerular microvascular endothelial cells
NF-κB: Nuclear factor kappa B
SIRT1: Sirtuin 1
8-oxoG: 8-Oxoguanine
cGAS: Cyclic GMP-AMP synthase
TLR4: Toll-like receptor 4
NOX2: NADPH oxidase 2
MtFt: Mitochondrial ferritin
MFRN1/2: Mitoferrin 1/2
LIP: labile iron pool
GPX4: glutathione peroxidase 4
PUFAs: polyunsaturated fatty acids
PL-OOH: phospholipid hydroperoxide
GSDMD: cleavage Gasdermin D
NCOA4: Nuclear receptor coactivator 4
FTH1: Ferritin heavy chain 1
eGFR: Estimated glomerular filtration rate
KD: Ketogenic diet
DN: Diabetic nephropathy
βOHB: β-Hydroxybutyric acid
SHR: Spontaneously hypertensive rat
PLADO: Plant Low-Protein Diet
ESCC: esophageal squamous cell carcinoma
